# Enhancement of Macarpine Production in *Eschscholzia Californica* Suspension Cultures under Salicylic Acid Elicitation and Precursor Supplementation

**DOI:** 10.3390/molecules25061261

**Published:** 2020-03-11

**Authors:** Andrea Balažová, Júlia Urdová, Vladimír Forman, Pavel Mučaji

**Affiliations:** 1 Department of Cell and Molecular Biology of Drugs, Faculty of Pharmacy, Comenius University, Kalinčiakova 8, Bratislava 83232, Slovakia; 2 Department of Pharmacognosy and Botany, Faculty of Pharmacy, Comenius University, Odbojárov 10, Bratislava 83232, Slovakia; urdova5@uniba.sk (J.U.); forman@fpharm.uniba.sk (V.F.)

**Keywords:** Macarpine, Sanguinarine, *Eschscholzia californica*, Salicylic acid, L-tyrosine, CYP719A2/A3, 3’-hydroxy-N-methyl-*(S)*-coclaurine 4′-O-methyltransferase

## Abstract

Macarpine is a minor benzophenanthridine alkaloid with interesting biological activities, which is produced in only a few species of the Papaveraceae family, including *Eschscholzia californica*. Our present study was focused on the enhancement of macarpine production in *E. californica* suspension cultures using three elicitation models: salicylic acid (SA) (4; 6; 8 mg/L) elicitation, and simultaneous or sequential combinations of SA and L-tyrosine (1 mmol/L). Sanguinarine production was assessed along with macarpine formation in elicited suspension cultures. Alkaloid production was evaluated after 24, 48 and 72 h of elicitation. Among the tested elicitation models, the SA (4 mg/L), supported by L-tyrosine, stimulated sanguinarine and macarpine production the most efficiently. While sequential treatment led to a peak accumulation of sanguinarine at 24 h and macarpine at 48 h, simultaneous treatment resulted in maximum sanguinarine accumulation at 48 h and macarpine at 72 h. The effect of SA elicitation and precursor supplementation was evaluated also based on the gene expression of 4′-OMT, CYP719A2, and CYP719A3. The gene expression of investigated enzymes was increased at all used elicitation models and their changes correlated with sanguinarine but not macarpine accumulation.

## 1. Introduction

Macarpine is a minor benzophenanthridine alkaloid produced only in a few species of the Papaveraceae family such as *Macleya microcarpa*, *Stylophorum lasiocarpum* [1] and *Eschscholzia californica* [2]. Macarpine shares a common biosynthetic route with sanguinarine, in which dihydrosanguinarine represents the branch point in the biosynthesis of both alkaloids. While dihydrosanguinarine undergoes the conversion to sanguinarine in a single-step reaction catalysed by dihydrobenzophenanthridine oxidase [3], the formation of macarpine requires five additional reactions, including two hydroxylations, two methylations, and the final oxidation. The macarpine biosynthetic pathway contains another branching point at dihydrochelirubine that is diverted to the formation of chelirubine [4]. Macarpine represents the most highly oxidized compound among benzophenanthridine alkaloids and its biosynthetic pathway is one of the longest that has been completely elucidated at the enzyme level [5,6], but the genes involved in the macarpine biosynthetic branch have not been fully characterized [7,8].

In general, quaternary benzophenanthridine alkaloids possess a broad spectrum of biological activities, including anti-bacterial, anti-viral, anti-oxidative, anti-inflammatory and neuroprotective properties [9,10,11]. Recently, the strong antiproliferative and apoptotic effects of sanguinarine, chelerythrine, macarpine and chelirubine against a broad spectrum of human cancer cell lines have been identified, which predetermine their use as an adjuvants in cancer therapy [12,13,14]. Besides the mentioned activities, macarpine evinces an ability to interact with DNA and its fluorescence properties could be used as a DNA probe for fluorescence microscopy and flow cytometry including analyses of the cell cycle [1]. Although two chemical synthesis of macarpine have been published in 1995 [15] and 2018 [16], no commercial macarpine is available to date. The low production of macarpine in natural sources limits its isolation in desired amounts as well as the biological activity testing. On the other hand, the natural overproduction of benzophenanthridine alkaloids in plants is related to the defence processes against various sorts of phytopathogens [17,18]. This fact is fundamental for the elicitor-triggered production of benzophenanthridines in cell cultures of natural producers [19,20,21]. Elicitor-enhanced production of macarpine in plants could be a way to acquire a more significant amount of this alkaloid, and thus to extend the portfolio of its biological activities. The present study is therefore focused on the demonstration of the elicitation effect of salicylic acid (SA) on the production of sanguinarine and macarpine in suspension cultures of *E. californica* supplemented by L-tyrosine as a precursor of benzophenanthridine alkaloids.

The effect of salicylic acid elicitation and precursor supplementation was investigated on two levels—alkaloid production and gene expression of enzymes involved in the biosynthesis of sanguinarine and macarpine—3′-hydroxy-N-methyl-(S)-coclaurine 4′-O-methyltransferase (4′-OMT; EC 2.1.1.116), (S)-stylopine synthase 1 (CYP719A2; EC 1.14.19.64), and trifunctional (S)-stylopine synthase 2/(S)-nandinine synthase/(S)-canadine synthase (CYP719A3; EC 1.14.19.73). 4′-OMT was selected due to its catalytic function in the pre-reticuline pathway of benzophenanthridine alkaloid biosynthesis as well as considerable overexpression under stress conditions [22]. Cytochrome P450 enzymes CYP719A2 and CYP719A3 contribute to a methylenedioxy bridge formation in the molecule of benzophenanthridine alkaloids [23].

## 2. Results

### 2.1. Alkaloid Production Pattern in Suspension Cultures under Elicitation

**Figure 1:**
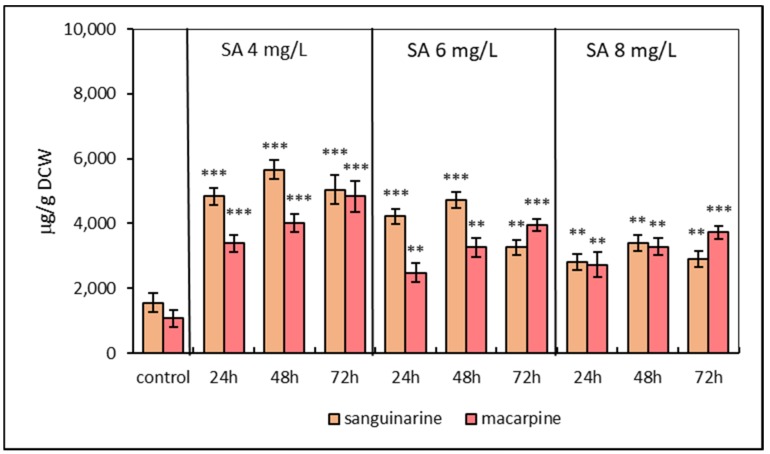
Production of sanguinarine and macarpine in suspension cultures of *E. californica* elicited with salicylic acid (SA) in time and dose-dependent manner. Values represent means ± SD from triplicate samples of three parallel experiments. The chart displays statistically significant differences (** *p* ≤ 0.01, *** *p* ≤ 0.001) of sanguinarine and macarpine content between non-elicited (control) and elicited samples.

*Eschscholzia californica* suspension cultures were subjected to the SA elicitation at three concentrations (4, 6, 8 mg/L). The effect of the elicitor was evaluated based on the production of benzophenanthridine alkaloids sanguinarine and macarpine. TLC separation of methanolic extracts and subsequent UV detection displayed an increased fluorescence of spots corresponding to sanguinarine and macarpine in comparison to the non-elicited cultures. The fluorometric quantifications of isolated alkaloids confirmed their increased production/accumulation in the phytomass of elicited *E. californica*. SA stimulated the production of both investigated alkaloids at all used concentrations (Figure 1). Regarding the production/accumulation of alkaloids, the most effective was the concentration of SA at 4 mg/L. Sanguinarine accumulation in SA elicited suspension cultures showed an upward trend with a maximum at 48 h and declined thereafter. Sanguinarine reached the maximum content of 5665.22 ± 435.8 μg/g dried cell weight (DCW) 48 h after SA elicitation at concentration 4 mg/L. In comparison to the sanguinarine production, macarpine was produced linearly over 72 h lasting elicitation with a maximum value of 4839.16 ± 486.3 μg/g DCW in SA-elicited cultures at 4 mg/L of SA. Overall, the production of sanguinarine and macarpine increased 3- and 4.5-fold, respectively, compared to non-elicited samples.

**Figure 2:**
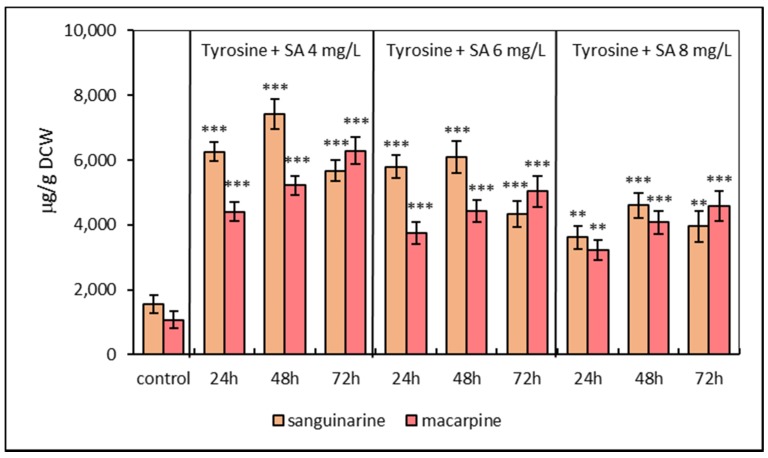
Production of sanguinarine and macarpine in simultaneously elicited suspension cultures of *E. californica* with salicylic acid (SA) and L-tyrosine (1 mM) in a time and dose-dependent manner. Values represent means ± SD from triplicate samples of three parallel experiments. The chart displays statistically significant differences (** *p* ≤ 0.01, *** *p* ≤ 0.001) of sanguinarine and macarpine content between non-elicited (control) and elicited samples.

The second elicitation model was based on the simultaneous treatment of *E. californica* suspension cultures with SA (4, 6, 8 mg/L) and L-tyrosine (1 mmol/L). Simultaneous elicitation exhibited a similar time- and dose-dependent manner in sanguinarine and macarpine production. The maximal sanguinarine level of combined elicitation with L-tyrosine and SA at concentration 4 mg/L was detected after 48 h (7421.44 ± 489.2 μg/g DCW) and was 4.7-times greater in comparison with control. The macarpine content was 6290.91 ± 470.7 μg/g DCW (six times higher than non-elicited cultures) after 72 h of treatment with the same elicitor combination. Compared to SA elicitation, the presence of L-tyrosine in growth media increases both sanguinarine and macarpine content from 30% to 40% (Figure 2).

**Figure 3:**
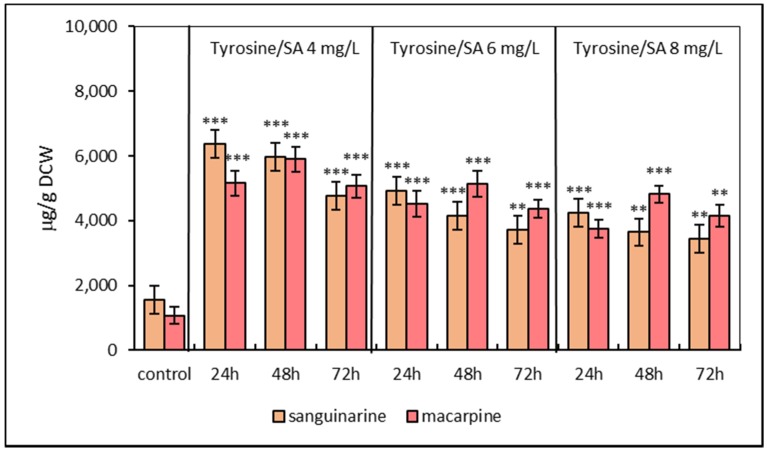
Production of sanguinarine and macarpine in sequential elicited suspension cultures of *E. californica* with SA and L-tyrosine (1 mmol/L) in a time and dose-dependent manner. Values represent means ± SD from triplicate samples of three parallel experiments. The chart displays statistically significant differences (** *p* ≤ 0.01, *** *p* ≤ 0.001) of sanguinarine and macarpine content between non-elicited (control) and elicited samples.

The third elicitation model included pre-treatment of suspension cultures with L-tyrosine for 24 h followed by SA elicitation. This model showed differences in alkaloid accumulation in suspension cultures. The simultaneous application of L-tyrosine and SA (4, 6, 8 mg/L) resulted in an increase in sanguinarine and macarpine after 48 and 72 h, respectively. Sequential treatment led to a peak accumulation in both alkaloids at 24 (sanguinarine) and 48 h (macarpine), respectively (Figure 3). The most effective concentration of SA regarding the maximal production/accumulation of sanguinarine and macarpine was 4 mg/L.

### 2.2. Gene Expression Assessment

**Figure 4:**
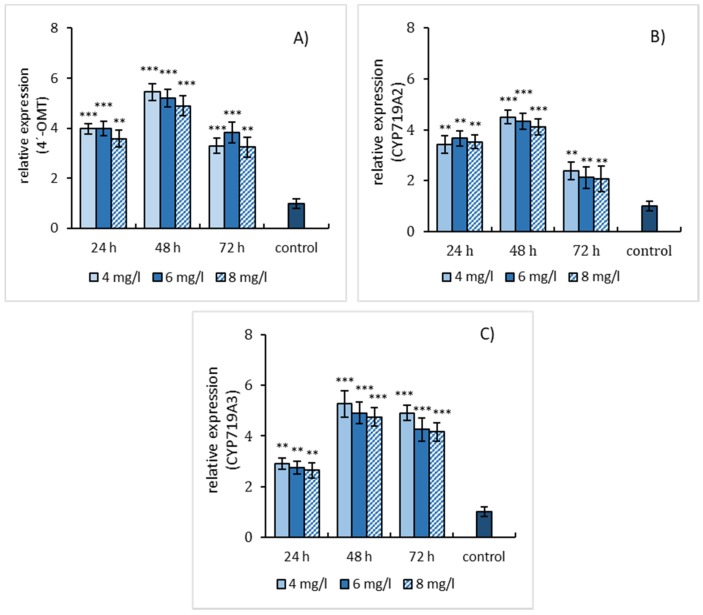
Salicylic acid-induced expressions of 4′-OMT (**A**), CYP719A2 (**B**), and CYP719A3 (**C**) genes in *E. californica* suspension cultures. The relative expression level shows values standardized by that of the non-elicited sample (control) as 1. Values are means ± SD from triplicate samples of three parallel experiments. Charts display statistically significant differences (** *p* ≤ 0.01, *** *p* ≤ 0.001) of gene expressions between non-elicited (control) and elicited samples.

The effect of three elicitation models was also evaluated concerning the relative gene expression of enzymes involved in the biosynthesis of sanguinarine and macarpine. The gene expression of three enzymes, 4′-OMT, CYP719A2, and CYP719A3, was evaluated in elicited suspension cultures. Simple SA elicitation of *E. californica* suspension cultures caused an increase in relative gene expression of 4′-OMT, CYP719A2, and CYP719A3 within 48 h of treatment and declined at longer-lasting elicitation. Notably, 4′-OMT (Figure 4A) and CYP719A3 (Figure 4C) showed a higher relative gene expression (~5 times compared to the non-elicited cultures) than CYP719A2 (Figure 4B).

**Figure 5:**
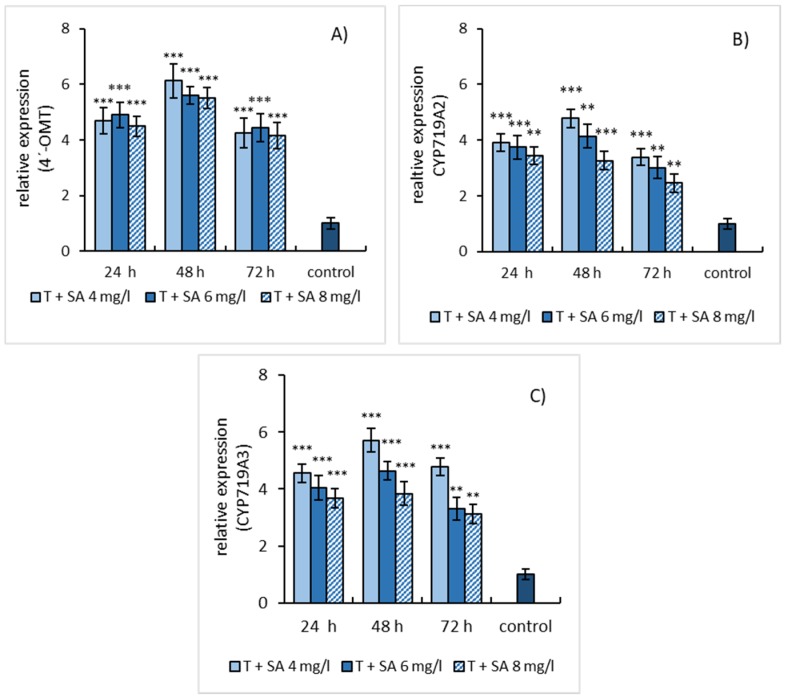
Expression patterns of 4′-OMT (**A**), CYP719A2 (**B**), and CYP719A3 (**C**) genes in *E. californica* suspension cultures affected by the combination of SA and L-tyrosine (1 mmol/L). The relative expression level shows values standardized by that of the non-elicited (control) sample as 1. Values are means ± SD from triplicate samples of three parallel experiments. Charts display statistically significant differences (** *p* ≤ 0.01, *** *p* ≤ 0.001) of gene expressions between non-elicited (control) and elicited samples.

Simultaneous application of SA and L-tyrosine induced the gene expression of estimated enzymes also and resulted in the same expression kinetics as SA elicitation. Although, the gene expression profiles of both elicitation models evinced a similar trend, at simultaneous elicitation a higher relative gene expression of enzymes was detected than in cases when only SA was used. This growth in relative gene expression can be assigned to the effect of L-tyrosine (Figure 5A–C).

**Figure 6:**
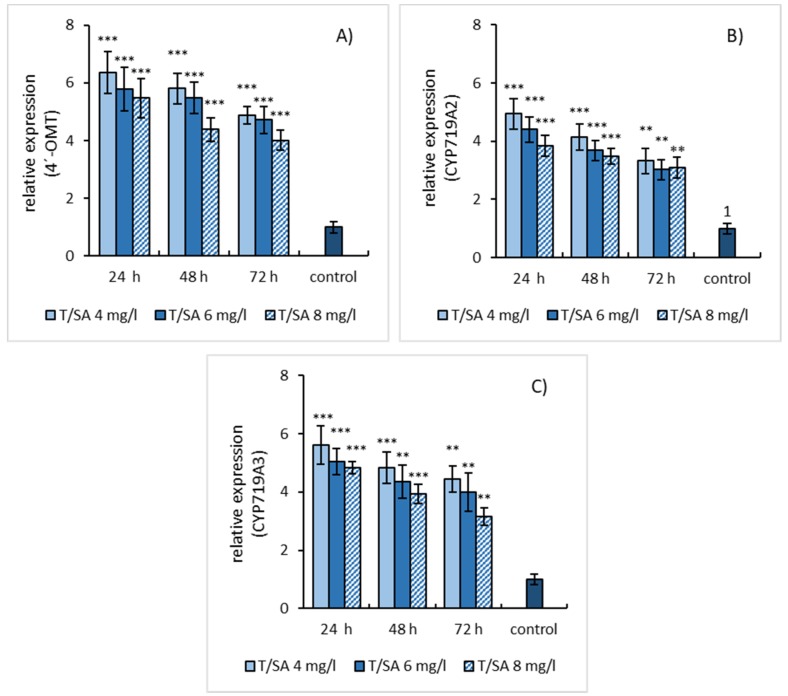
Expression patterns of 4′-OMT (**A**), CYP719A2 (**B**), and CYP719A3 (**C**) genes in *E. californica* suspension cultures pre-treated by L-tyrosine (1mmol/L) for 24 h followed by SA elicitation. The relative expression level shows values standardized by that of non-elicited (control) sample as 1. Values are means ± SD from triplicate samples of three parallel experiments. Charts display statistically significant differences (** *p* ≤ 0.01, *** *p* ≤ 0.001) on gene expressions between non-elicited (control) and elicited samples.

Sequential treatment of suspension cultures with L-tyrosine 24 h before SA elicitation caused distinct relative gene expression of studied enzymes with the maximum at 24 h and its graduated reduction within 72 h (Figure 6A–C). The shift in gene expression toward a shorter elicitation time probably results from the induction effect of L-tyrosine.

Expression pattern of 4′-OMT, CYP719A2, and CYP719A3 in the case of all elicitation models correlated with sanguinarine but not with macarpine accumulation.

## 3. Discussion

Benzophenanthridine alkaloid macarpine is produced only in limited numbers of plant species and studied to a lesser extent than sanguinarine and chelerythrine [24]. The biosynthetic pathway of macarpine has been completely elucidated in plants at the enzyme level. The genes involved in sanguinarine formation are completely identified, whereas the nucleotide sequences of enzymes catalyzing the last steps of macarpine biosynthesis are clarified only partially [5,6,8]. Sanguinarine falls into the category of phytoalexins that protects plants against phytopathogens and prevents the development of diseases. Besides sanguinarine, other benzophenanthridines, such as chelerythrine, contribute to plant defence response. It might be supposed that these major compounds sufficiently protect plants against stress [22], therefore the formation of macarpine in the longest biosynthetic route remains at a minor level. However, if the production of sanguinarine is inducible by various exogenous elicitors that mimic the pathogen attack (biotic elicitation), the production of macarpine in intact plants or cell cultures of natural producers is also increased. Recent studies have revealed the prospective biological activities of macarpine toward cancer cells [12,14,24], but extensive testing is limited by its availability from natural sources.

The benzophenanthridine alkaloid production in plant species of Papaveraceae family has been evaluated under diverse elicitor treatments [20,21,25,26]. In the majority, the effect of elicitors has been assessed in relation to sanguinarine and chelerythrine content. Elicitors related to the signal transduction pathways such as methyl jasmonate (MJ), SA, fungal extracts, as well as their combinations, resulted in increased production of sanguinarine in plants [22,23,25,27].

Our experiments were focused on the evaluation of the SA elicitation on the sanguinarine and macarpine accumulation in suspension cultures of *E. californica* that was supported by the supplementation of L-tyrosine. Under simple SA elicitation, the sanguinarine content reached a maximum at 48 h, while the macarpine level gradually increased over 72 h. SA exhibited the most efficient elicitation effect at concentrations of 4 mg/L. Higher doses of SA (6 and 8 mg/L) stimulated sanguinarine and macarpine production/accumulation less effectively. Nevertheless, the content of both alkaloids was increased in comparison to non-elicited cultures. A similar alkaloid accumulation profile was also identified in the second elicitation model consisting of simultaneous dosage of SA and L-tyrosine in growth media. The third elicitation model was based on the sequential addition of SA 24 h after L-tyrosine supplementation of suspension cultures. Accumulation of sanguinarine reached the maximum at 24 h and macarpine at 48 h. L-tyrosine supplementation in the case of the second and third elicitation models contributed to the increase in sanguinarine and macarpine levels from 30% to 40%. The effect of tyrosine feeding with fungal elicitor treatment has been investigated in *E. californica* suspension cultures. This approach resulted in the growth of total alkaloid content at the late exponential stage, but the feeding of tyrosine without elicitation did not enhance alkaloid formation in *E. californica* significantly [28]. Tyrosine and shikimate supplementations of in vitro cultures of *Papaver somniferum* have resulted in no significant changes in sanguinarine production as well. On the other hand, when *Trichoderma harzianum* culture filtrate was combined with shikimate, the highest sanguinarine production in *Papaver somniferum* cultures was achieved [29]. Another study has evaluated the tyrosine feeding on the formation of phenylethanoid glycosides in *Cistanche deserticola* cell suspension culture [30]. Cho et al. 2008 have studied the effect of sequential elicitation with MJ, SA and yeast extract on the formation of benzophenanthridine alkaloid in *E. californica*. In the experiment, the SA was used at a concentration of 1.5 mg/L. The obtained results confirmed the synergic effect of elicitors that enhanced sanguinarine and dihydrosanguinarine production 5.5- and 2.5-fold respectively [27]. Similar results to those of Cho et al. 2008 have been published in the article of Kollárová et al. 2014. This study assessed the alteration of sanguinarine production and LOX activity in *E. californica* suspension cultures subjected to simple SA elicitation at the concentration of 1.5 mg/L [25]. Based on these findings, it can be concluded that low concentrations of SA elicit more efficiently the formation of benzophenanthridines in *E. californica* in vitro cultures. Among abiotic elicitors, manganese chloride at the concentration of 10 mg/L exhibits a notable effect on macarpine production in *E. californica* suspension cultures [21]. Based on these findings, it can be concluded that our results correspond with the outcomes reported in the above-mentioned studies.

The production of benzophenanthridine alkaloids and their dihydro forms has been extensively evaluated in *E. californica* cultured cells after yeast elicitor treatment in the article of Weiss et al. 2006. HPLC analysis revealed an increased accumulation of benzophenanthridines, mainly macarpine and chelirubine, in the outer medium of cell cultures, whereas their dihydro forms were identified in cells. On the other hand, the external addition of sanguinarine in the growth media of *E. californica* cell cultures resulted in rapid absorption into cells and its subsequent conversion to less toxic dihydrosanguinarine by sanguinarine reductase. The existence of this conversion mechanism can avoid the cytotoxic effect of sanguinarine on producing cells. The sequence of the gene and protein of sanguinarine reductase isolated from *E. californica* suspension cultures as well as its catalytic mechanism have been published in the article of Vogel et al. 2010 [4,31].

In the present study, the effect of SA and precursor supplementation was evaluated also from the perspective of the gene expression of three enzymes involved in the biosynthesis of sanguinarine and macarpine. 4′-OMT is engaged in the pre-reticuline pathway of benzophenanthridine alkaloid biosynthesis whose protein/gene overexpression has been confirmed under elicitor treatment [23,27,32,33]. Two cytochrome P450 enzymes CYP719A2, and CYP719A3 are involved in the biosynthesis of benzophenanthridine alkaloids that possess the ability to form methylenedioxy bridge in the molecule of cheliantifoline. While CYP719A2 converts cheliantifoline to stylopine, CYP719A3 exhibits an ability to convert (S)-scoulerine to (S)-nandinine and (S)-tetrahydrocolumbamine to (S)-canadine. More extensive substrate specificity of CYP719A3 predetermines its engagement in the biosynthesis of another structural subgroup of benzylisoquinoline alkaloids [23].

As results show that, at SA elicitation, the relative gene expression of all estimated enzymes exhibited similar time- and dose-dependent profiles as sanguinarine production in suspension cultures but differ from the profile of macarpine production. QRT-PCR revealed a more than 5-fold increase in gene expression of 4′-OMT and CYP 719A3 and 4-fold increase of CYP719A2 gene expression. Simultaneous application of SA and L-tyrosine resulted in the same gene expression profiles of enzymes with higher values of relative gene expression as the simple SA elicitation. The elevation of relative gene expression can be assigned to the synergic effect of L-tyrosine supplementation and SA elicitation. Distinct relative gene expression patterns were detected in the case of sequential treatment of suspension cultures of *E. californica* with SA and L-tyrosine that reached the maximum at 24 h of elicitation. The relative gene expression of all estimated enzymes increased to a similar level as at the simultaneous treatment. Among the studied enzymes, 4′-OMT and CYP719A3 exhibit a higher gene expression intensity than CYP719A2. Higher values of CYP719A3 gene expression compared to CYP719A2 result from its trifunctional character, which enables the involvement of CYP719A3 in biosynthetic pathways of other benzylisoquinoline alkaloid subgroups. The coordinated induction of both CYP719A2 and CYP719A3 genes has been discovered in *E. californica* seedlings treated with MJ as well [23]. The effect of MJ, salicylic acid and yeast extract on the protein expression O 4′-OMT, 6-OMT, BBE, CYP70B1, and DHBO has been investigated in suspension cultures of *E. californica* [27,32]. The results showed variations in protein expression profiles of tested enzymes in relation to sanguinarine and dihydrosanguinarine production. Increased gene expression of enzymes involved in sanguinarine biosynthesis has been also found in *Papaver somniferum* suspension cultures subjected to elicitation with MJ and fungal elicitors [29,33]. Based on the outcomes of the present study, it can be concluded that precursor feeding in combination with SA elicitation effectively stimulates the production of sanguinarine and macarpine. The gene expression of biosynthetic enzymes in *E. californica* suspension cultures provided detailed information about the bioprocessing of alkaloids in plants under elicitation.

## 4. Materials and Methods

### 4.1. Preparation of Plant Material

Suspension cultures of *E. californica* were prepared from friable callus cultures, keeping the conditions as published previously in the study by Balažová et al. 2018 [21].

### 4.2. Elicitor Preparation and Elicitation Models

Three elicitation models were used for the elicitation of *E. californica* suspension cultures. Simple salicylic acid elicitation (SA) at final concentrations of 4, 6, and 8 mg/L in growth media, the model included combinations of SA (4, 6, 8, mg/L) with L-tyrosine at final concentration 1 mM simultaneously added into growth media and pre-treatment of suspension cultures with L-tyrosine (1 mmol/L) 24 h before salicylic acid elicitation (sequential treatment).

Stock solutions were prepared by dissolution of 0.4 g of salicylic acid (Merck, Germany) and 0.575 g of L-tyrosine hydrochloride (Sigma-Aldrich, St. Louis, MS, USA) in 100 mL of sterile distilled water. Adequate volumes of both stock solutions were aseptically dosed to the growth media of suspension cultures through 0.22 µm syringe membrane filters (Millipore, Merck, Germany) to achieve the desired concentrations. The presence of an elicitor and precursor were omitted in control samples. All samples were prepared in triplicate. Suspension cultures were maintained on the orbital rotator (110 rpm) at 24 °C and relative humidity of 70%. Plant material was harvested 24, 48 and 72 h after elicitor treatment. Phytomass was finally separated from growth medium by vacuum filtration, lyophilized and stored at −20 °C.

### 4.3. Isolation and Purification of Macarpine

Macarpine (as a reference standard) was isolated from the methanolic extract of *E. californica* suspension cultures by column chromatography (30 × 1.5 cm) on Silicagel 60 Å (Merck, Germany) using chloroform:methanol:benzene (14:3:3 by vol.) as mobile phase. The elution of fractions containing macarpine was monitored by a UV at 366 nm. The combined macarpine fractions were evaporated to dryness and subsequently dissolved in distilled water. A pH of the water solution was adjusted to 9–10 by 1–2 drops of ammonia and extracted with chloroform. The chloroform fraction was finally evaporated to the dryness. The macarpine obtained by this procedure was subsequently subjected to ^1^H-NMR analysis (Varian NMR System 600, Santa Clara, CA, USA, supplementary data Figure S1), identity [34] and purity verification. Purified macarpine was used as a reference standard in fluorescence assay.

### 4.4. Isolation and Quantification of Alkaloids

Benzophenanthridine alkaloids sanguinarine and macarpine were isolated from 0.5 g of lyophilized plant material suspended in 10 mL of methanol. Extraction was performed overnight on the orbital rotator (110 rpm) followed by centrifugation at 10,000 g for 15 min. Each extracted sample (50 μL) was analysed by TLC (Silicagel 60 plates, Merck, Germany) using chloroform:methanol:benzene (12:7:1 by vol.) developing system. TLC plates were subsequently subjected to UV detection at 366 nm (Camag Reprostar II). Macarpine and sanguinarine were identified based on comparison with reference standards. Commercial sanguinarine was purchased from Sigma (St. Louis, MS, USA) and macarpine as a reference standard was isolated from the methanolic extract of *E. californica*.

Sanguinarine and macarpine spots were separately removed from the TLC plate and extracted in 1 mL of 50% ethanol for UV spectroscopy containing 0.02 mol/L NaOH for 1 h. After centrifugation at 6000 rpm for 15 min, volumes of supernatants were adjusted to 10 mL by 50% ethanol for UV spectroscopy containing 0.02 mol/L NaOH.

Isolated benzophenanthridine alkaloids were quantified on the base of their fluorescence at λex/em = 324/408 nm for sanguinarine [25] and λex/em = 269/420 nm for macarpine [21] (Supplementary data Figures S2 and S3). Final alkaloid concentration was calculated according to the fluorescence intensity of reference standards and expressed as μg/g dried cell weight (DCW). Calibration solutions of reference standards were prepared in the concentration range from 25–100 μg/L with r^2^ = 0.999 for the sanguinarine and r^2^ = 0.997 for macarpine calibration curve.

### 4.5. Isolation of Total RNA and Quantitative RT-PCR

The isolation of total RNA was realized according to the procedure published in the article Balažova et al. 2018 [21]. Briefly, RNAzol RT (Sigma-Aldrich, SR) was used for isolation of total RNA from 100 mg of lyophilized cell culture. RNA was converted to cDNA followed the PrimeScript RT Reagent Kit (Takara, Japan) manufacturing protocol. Amplification and detection of reference and target gene cDNA were performed on a 7300 Real-Time PCR System (Applied Biosystems, Singapore) using HOT FIREPol EvaGreen qPCR Mix Plus (ROX) (Solis BioDyne, Tartu, Estonia). PCR products were evaluated on the base of the melting curve analysis to confirm the specific amplification. Relative expression of 4′-OMT, CYP719 A2, and CYP719A3 was calculated using the ΔΔCt value method [35]. As a reference gene, β-actin was used. Nucleotide sequences of primers were acquired from the article of Ikezawa et al. 2007 [23] and are listed in Table 1.

### 4.6. Statistical Analysis

Experiments were performed in triplicate and data were expressed as means ± standard deviations. Independent samples Student t-test was used to determine significant (*p* ≤ 0.05) differences between the non-elicited (control) and elicited samples. The statistical relationship between the gene expression of investigated enzymes and alkaloid production in elicited cultures was evaluated according to Pearson’s correlation test.

## 5. Conclusions

The present study evaluates the effect of various concentrations of salicylic acid and L-tyrosine supplementation on the production of sanguinarine and macarpine in *E. californica* suspension cultures. The evaluated elicitation models exhibit a significant ability to improve macarpine and sanguinarine production. The most promising results were obtained using SA elicitation (4 mg/L) in combination with L-tyrosine added either simultaneously or sequentially into growth media. Besides alkaloid production, the increased relative gene expression of 4′-OMT, CYP719A2, and CYP719A3 was confirmed. Alterations in the gene expression of studied enzymes showed the correlation with sanguinarine but not macarpine production. The investigation of gene expression of biosynthetic enzymes provides detailed information about the bioprocessing of alkaloids in plants subjected to different biotechnological approaches. Additionally, the macarpine purification procedure seems to be efficient for its isolation from *E. californica* in the desired amount. In conclusion, salicylic acid elicitation supported by L-tyrosine seems to be a promising approach in the improvement in macarpine production in natural sources.

## Figures and Tables

**Table 1:** Sequences of primers used for qRT-PCR.

Primer Name	Oligonucleotide Sequences (5′- to 3′-)
4′-OMT	forward CCTAGAAGAGGAATCAGAACATCCA reverse TCACTTCTCTCCCTTCCACCA
CYP719A	forward GTCGTAATTAATCACTTAACCGTGCTCG reverse GAAAGAAACAGAGCAAATCTTATCCTTTTACC
CYP719A3	forward CCTCGTAACTAATATACCAGTGTGGTG reverse GACAACCAAGCAAACTCTTATTCTTGTAC
β-actin	forward GGTATTGTGCTGGATTCTGGTG reverse GTAGGATTGCGTGGGGTAGTG

## Supplementary Materials

The following are available online at https://www.mdpi.com/1420-3049/25/6/1261/s1. Figure S1: ^1^H NMR spectrum of macarpine. Figure S2: Excitation (A) and emission (B) spectra of macarpine measured in the mixture of 50% ethanol for UV spectroscopy containing 0.02 mol/L NaOH. Figure S3: Excitation (A) and emission (B) spectra of sanguinarine measured in the mixture of 50% ethanol for UV spectroscopy containing 0.02 mol/L NaOH.
