# Supplementary material for: Enhancement of Macarpine Production in Eschscholzia Californica Suspension Cultures under Salicylic Acid Elicitation and Precursor Supplementation
Source: Molecules. 2020 Mar 11;25(6):1261. doi: 10.3390/molecules25061261 (PMC7143939; doi:10.3390/molecules25061261)
Supplement: Supplementary file 1 [file molecules-25-01261-s001.pdf]

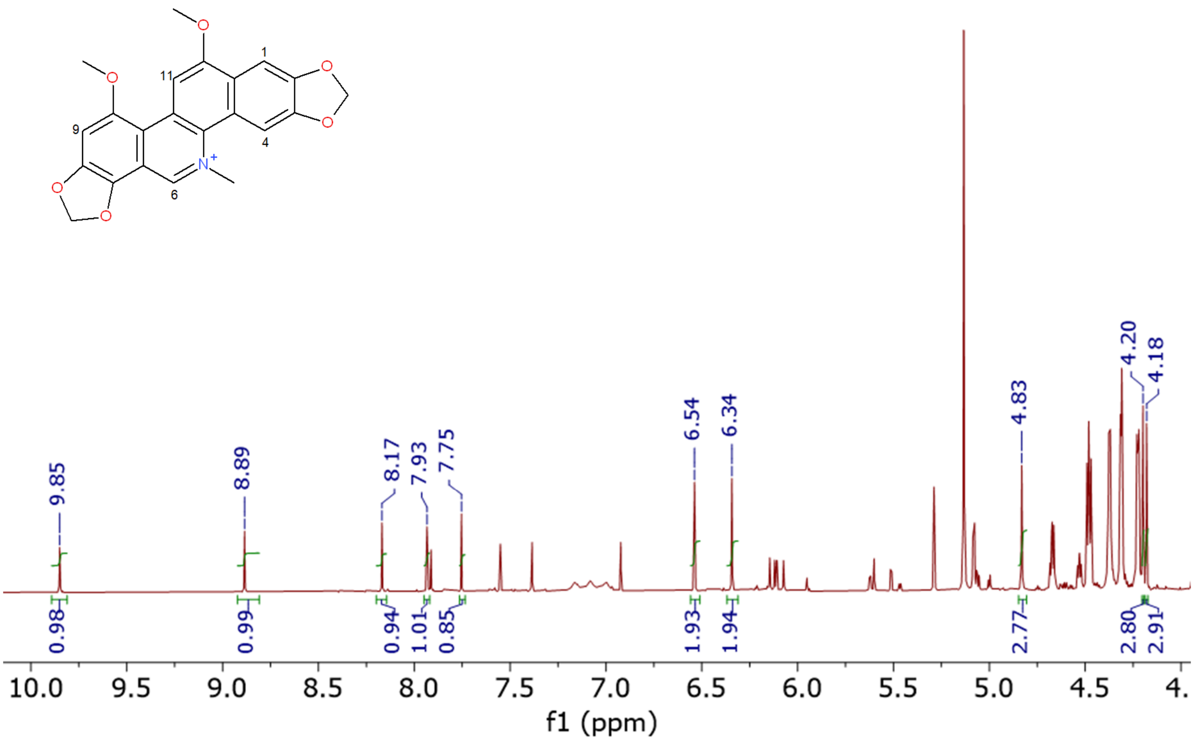

Figure S1: 1H NMR spectrum of macarpine.

1H NMR (600 MHz, DMSO-d6) δ 9.85 (s, 1H, H-C(6)), 8.89 (s, 1H, H-C(11)), 8.17 (s, 1H, H ar.), 7.93 (s, 1H, H ar.), 7.75 (s, 1H. H ar.), 6.54 (s, 2H,.OCH2O), 6.34 (s, 2H, OCH2O), 4.83 (s, 3H, CH3-N), 4.20 (s, 3H, CH3-O), 4.18 (s, 3H, CH3-O).

HRMS (ESI+): calcd. for [C22H17NO6+H]+ m/z 392.1129, found 392.1130

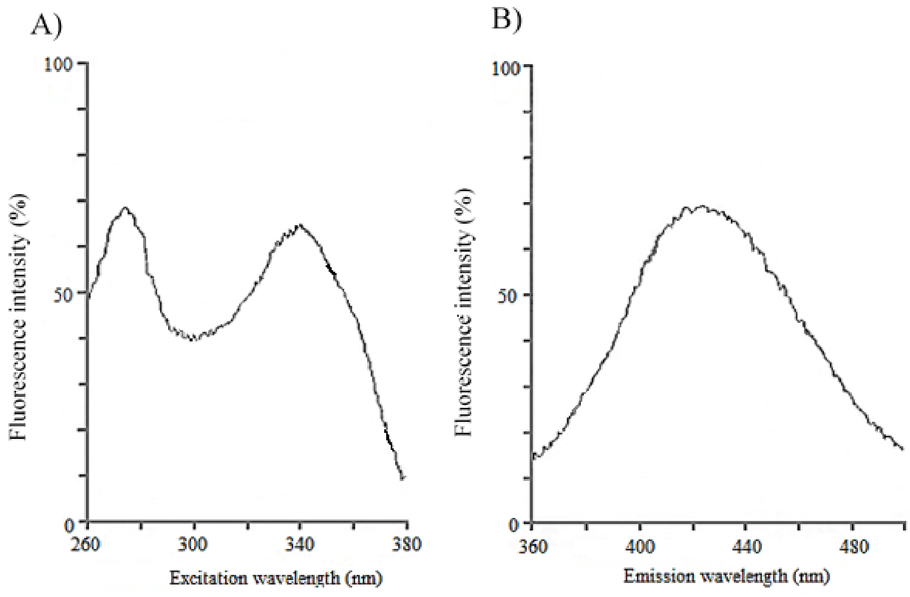

Figrue S2: Excitation (A) and emission (B) spectra of macarpine measured in the mixture of 50 % ethanol for UV spectroscopy containing 0.02 mol/l NaOH. Macarpine exhibited two peaks at 269 and 338 nm in the excitation spectrum and one single peak in the emission spectrum at 420 nm for excitation at 269 nm.

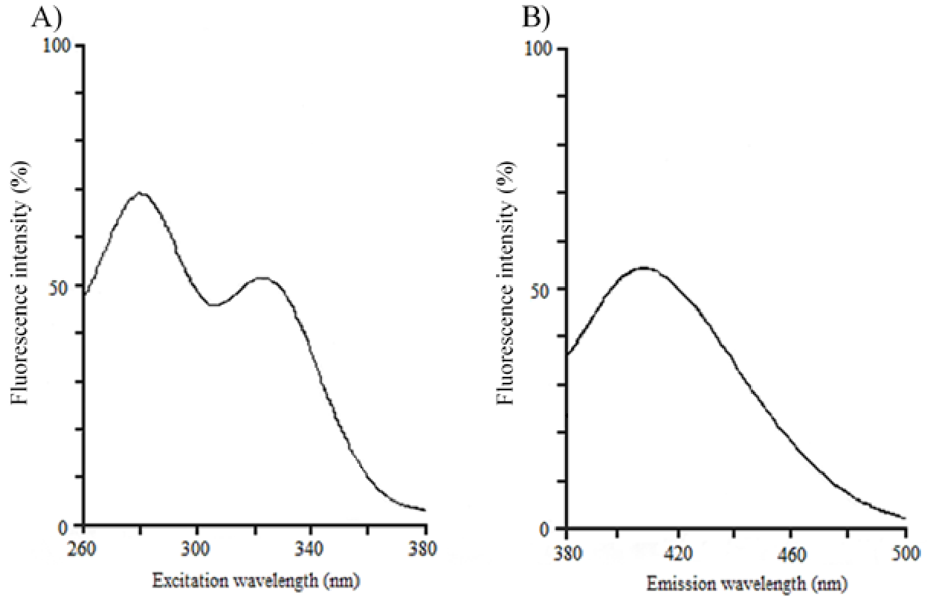

Figure S3: Excitation (A) and emission (B) spectra of sanguinarine measured in the mixture of 50 % ethanol for UV spectroscopy containing 0.02 mol/l NaOH. Sanguinarine exhibited two peaks at 280 and 324 nm in the excitation spectrum and one single peak in the emission spectrum at 408 nm for excitation at 324 nm.
